# Machine-Learning-Guided Experimental Prioritization of Seaweed-Derived Bioplastic Films Toward an LDPE-like Mechanical Target Using Sparse Literature Data

**DOI:** 10.3390/polym18172145

**Published:** 2026-09-02

**Authors:** Nicolas Rafael Andrés Gallardo Gatica, José Luis Valin Rivera, María Elena Fernández Abreu, Francisco Rolando Valenzuela Diaz, Meyli Valin Fernandez, Cristóbal Ignacio Galleguillos Ketterer, Daniel Francisco Leiva Palomera, Wanderley Ferreira de Amorim

**Affiliations:** 1 Escuela de Ingeniería Mecánica, Pontificia Universidad Católica de Valparaíso, Valparaiso 2340025, Chile; nicolas.gallardo.g@mail.pucv.cl (N.R.A.G.G.); maria.fernandez.a@pucv.cl (M.E.F.A.); cristobal.galleguillos@pucv.cl (C.I.G.K.); daniel.leiva.p@mail.pucv.cl (D.F.L.P.); 2 Departamento de Engenharia Metalúrgica e de Materiais, Escola Politécnica, Universidade de São Paulo, Av. Prof. Mello Moraes 2463, Sao Paulo 05508-010, SP, Brazil; 3 Department of Mechanical Engineering (DIM), Faculty of Engineering (FI), Universidad de Concepción, Concepcion 4030000, Chile; mvalin@udec.cl; 4 Mechanical Engineering Department, Federal University of Campina Grande, Campina Grande 58429-900, PB, Brazil; wanderley.ferreira@professor.ufcg.edu.br

**Keywords:** seaweed bioplastics, agar, alginate, carrageenan, machine learning, experimental prioritization, LDPE, mechanical properties, sparse data, repeated cross-validation

## Abstract

Seaweed polysaccharide films are potential alternatives to petroleum-derived flexible packaging, but literature data are sparse and heterogeneous. This study evaluates whether composition-only machine-learning models can prioritize reported seaweed formulations for experimental follow-up near a nominal low-density polyethylene (LDPE) mechanical target of 15 MPa tensile strength and 300% elongation at break. A published dataset of 115 formulations with 41 compositional predictors was analyzed using a regularized linear baseline (ridge regression), random forest, and a deliberately shallow, regularized XGBoost model. Model performance was estimated using repeated five-fold cross-validation (10 repeats; 50 test-fold evaluations). For tensile strength, mean *R*^2^ was 0.617 ± 0.174 for random forest and 0.628 ± 0.150 for XGBoost, with corresponding RMSE values of 12.35 ± 3.52 and 12.27 ± 3.84 MPa. For elongation, random forest and XGBoost reached mean *R*^2^ values of 0.498 ± 0.194 and 0.470 ± 0.188, respectively. The linear baseline was materially less stable, indicating that the available composition-property relations are not adequately represented by a global linear model. The maximum observed elongation was 172.5%, which is 42.5% below the 300% screening target; equivalently, the target lies 73.9% above the dataset maximum. Consequently, the model cannot establish LDPE equivalence or credible extrapolation to the target. Residual diagnostics, learning curves, and SHAP analyses were added to bound interpretation. The defensible outcome is experimental prioritization within the observed domain, not inverse design or material substitution.

## 1. Introduction

Flexible packaging materials must combine sufficient tensile resistance with deformation capacity, toughness, barrier performance, sealability, and stability under service humidity conditions. Seaweed-derived agar, alginate, and carrageenan are renewable film-forming polysaccharides, but their mechanical response is controlled by molecular structure, water content, plasticizer concentration, polymer blending, crosslinking, film-processing history, thickness, conditioning, and test protocol [1,2,3]. These factors make direct comparison among published formulations difficult.

Plasticizers illustrate the coupled physics. Glycerol and related low-molecular-mass compounds compete for intermolecular hydrogen-bonding sites, increase free volume and segmental mobility, and generally increase elongation while reducing modulus and tensile strength [4]. Water acts as an additional plasticizer in hydrophilic polysaccharide networks; therefore, relative humidity, conditioning time, and storage history can shift the apparent strength–ductility balance. The observed response is consequently not determined by composition alone.

Materials-informatics approaches can extract associations from the heterogeneous literature when prospective experiments are expensive [5,6,7]. The source database used here was compiled specifically to study sparse seaweed-bioplastic data and already demonstrated that tree-based algorithms can model parts of the reported composition-property relation [8]. A related agar-film dataset similarly shows a broad mechanical envelope among nominally comparable systems [9]. However, small-*n*, high-dimensional datasets are vulnerable to overfitting, unstable validation estimates, confounding by source study, and unsupported extrapolation [10,11].

The technical gap addressed here is therefore narrower than material “inverse design.” Existing work provides a composition-property database and predictive models, but it does not answer whether those records can be used as an evidence-bounded decision aid for prioritizing experiments near a declared LDPE-like tensile-elongation target while explicitly quantifying validation instability and target infeasibility. This study addresses that gap by (i) reframing the task as experimental prioritization rather than inverse design; (ii) replacing a single hold-out split with repeated five-fold cross-validation; (iii) adding a regularized linear baseline; (iv) reducing XGBoost depth and adding regularization; (v) adding residual, learning-curve, and SHAP diagnostics; and (vi) integrating physical interpretation of plasticization, humidity sensitivity, multicomponent interactions, and process dependence. The resulting claims are restricted to ranking within the observed literature domain.

## 2. Materials and Methods

### 2.1. Dataset, Scope, and Evidence Limits

The input contained 115 literature formulations, 41 compositional predictors, tensile strength σ (MPa), and elongation at break ϵ (%). Values were used as supplied after removal of the index column. No missing values or exact duplicate rows were found in the archived table. Tensile strength ranged from 0.249 to 79.53 MPa (median 10.50 MPa), and elongation ranged from 0 to 172.475% (median 17.40%). The predictor-to-sample ratio was 41/115 = 0.357, corresponding to 2.80 observations per predictor before resampling. This density is insufficient for unrestricted statistical inference and requires regularized models, resampling-based evaluation, and restricted claims.

The database aggregates measurements obtained under potentially different conditions. Relevant unrecorded variables include film thickness, relative humidity, conditioning duration, storage history, strain rate, gauge length, drying temperature, raw-material grade, concentration basis, and source laboratory. ASTM D882 provides a controlled framework for tensile testing of thin plastic sheeting, but not all corresponding fields are encoded in the present dataset [12]. Consequently, observed variance combines formulation effects, measurement uncertainty, and unmodeled inter-study effects. Source-paper identifiers were not available in the supplied table, so grouped cross-validation by publication could not be implemented; this remains a material limitation.

### 2.2. Models and Repeated Cross-Validation

Three regression models were evaluated for each response. The linear baseline was ridge regression after standardization of all 41 predictors, because the responses are continuous; logistic regression is not appropriate for tensile strength or elongation unless the problem is first converted into a categorical classification task. Random forest used 300 trees, square-root feature sampling, a minimum leaf size of two, and random state 42 [13]. XGBoost [14] was deliberately constrained relative to the original depth-4 configuration: 200 estimators, maximum depth 2, learning rate 0.03, subsample 0.8, column subsampling 0.8, *L*_2_ regularization λ=5, *L*_1_ regularization α=0.1, and random state 42. These settings were fixed before resampling; no hyperparameter search was performed on the evaluation folds.

Generalization performance was estimated with repeated five-fold cross-validation using 10 repeats and a fixed resampling seed, yielding 50 test-fold evaluations per model and target. The same fold assignments were used across models to preserve paired comparisons. Performance was summarized using the mean and standard deviation of

(1) $$R^{2}=1-\frac{\sum_{i}{\left(y_{i}-{\hat{y}}_{i}\right)}^{2}}{\sum_{i}{\left(y_{i}-\bar{y}\right)}^{2}},\;\mathrm{MAE}=n^{-1}\sum_{i}\left|y_{i}-{\hat{y}}_{i}\right|,\;\mathrm{RMSE}=\sqrt{n^{-1}\sum_{i}{\left(y_{i}-{\hat{y}}_{i}\right)}^{2}}.$$

The empirical 2.5th and 97.5th percentiles of fold scores were archived as descriptive resampling bounds. Because repeated folds are not statistically independent, these percentiles were not interpreted as formal confidence intervals. A deterministic shuffled five-fold split was additionally used to generate out-of-fold residual plots. Learning curves used the same five-fold scheme. These procedures reduce dependence on one 23-case hold-out but cannot replace external or source-grouped validation [11,15,16].

### 2.3. Model Interpretation

XGBoost models were refit to the full dataset only after validation for descriptive interpretation using SHAP [17]. Mean absolute SHAP values were used to rank global model attributions, and dependence plots were generated for the leading feature of each target. SHAP values describe the fitted model for the observed feature distribution; they are not causal effects and do not compensate for missing process, conditioning, molecular, or provenance variables.

### 2.4. Mechanical Similarity and Candidate Selection

The nominal screening coordinates were $\sigma_{t}$ = 15 MPa and $\epsilon_{t}$ = 300%. For each observed formulation, normalized distance and bounded similarity were computed as

(2) $$d_{i}=\sqrt{{\left(\frac{\sigma_{i}-\sigma_{t}}{\sigma_{t}}\right)}^{2}+{\left(\frac{\epsilon_{i}-\epsilon_{t}}{\epsilon_{t}}\right)}^{2}},\;S_{i}=\frac{1}{1+d_{i}}.$$

Equal weighting is an explicit screening choice rather than an application-specific utility function. The ranking was calculated from observed literature properties, not extrapolated predictions. A broad 10–20 MPa and 100–500% visualization window was used only as a reference. Mechanical equivalence would additionally require matched thickness, test direction, conditioning, modulus, toughness, tear and puncture resistance, water-vapor and oxygen transmission, thermal behavior, sealability, aging, migration, biodegradation conditions, and processability.

## 3. Results

### 3.1. Dataset Mechanical Envelope and Target Feasibility

**Figure 1:**
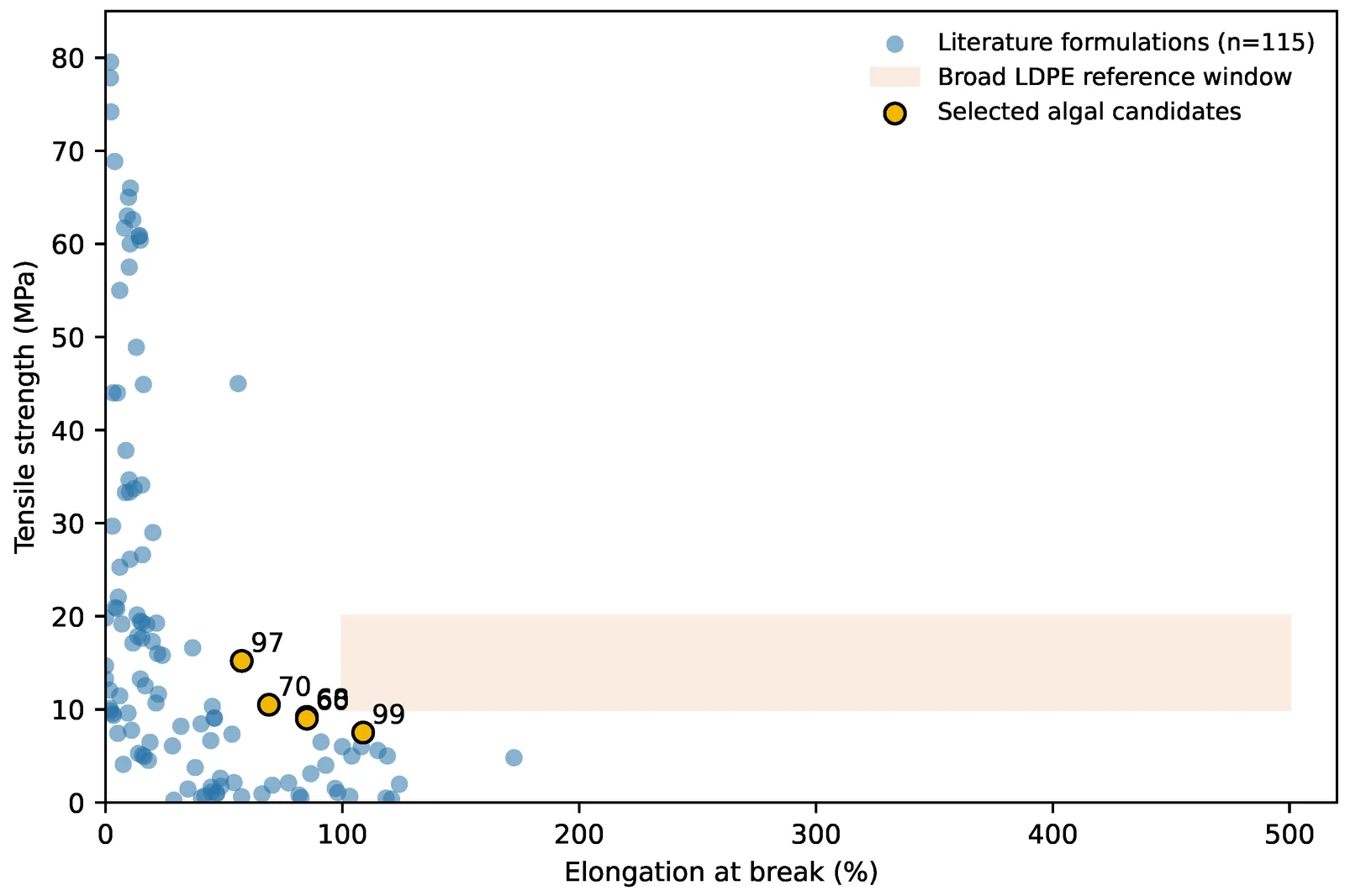
Mechanical map of the 115 literature formulations. The LDPE region is a screening reference rather than a specification or equivalence window. Candidate labels are dataset row identifiers.

Figure 1 shows that reported formulations overlap the nominal LDPE tensile region but not the adopted elongation target. The dataset maximum elongation is 172.475%. Expressed relative to the target, this maximum is 100(300 − 172.475)/300 = 42.5% below 300%. Expressed relative to the observed maximum, the target is 100(300 − 172.475)/172.475 = 73.9% above the maximum. The two percentages use different denominators and are therefore both correct. The key engineering point is unchanged: the target lies outside the observed response envelope, so tree ensembles cannot support long-range extrapolation to 300% elongation.

### 3.2. Repeated Cross-Validation and Linear Baseline

**Figure 2:**
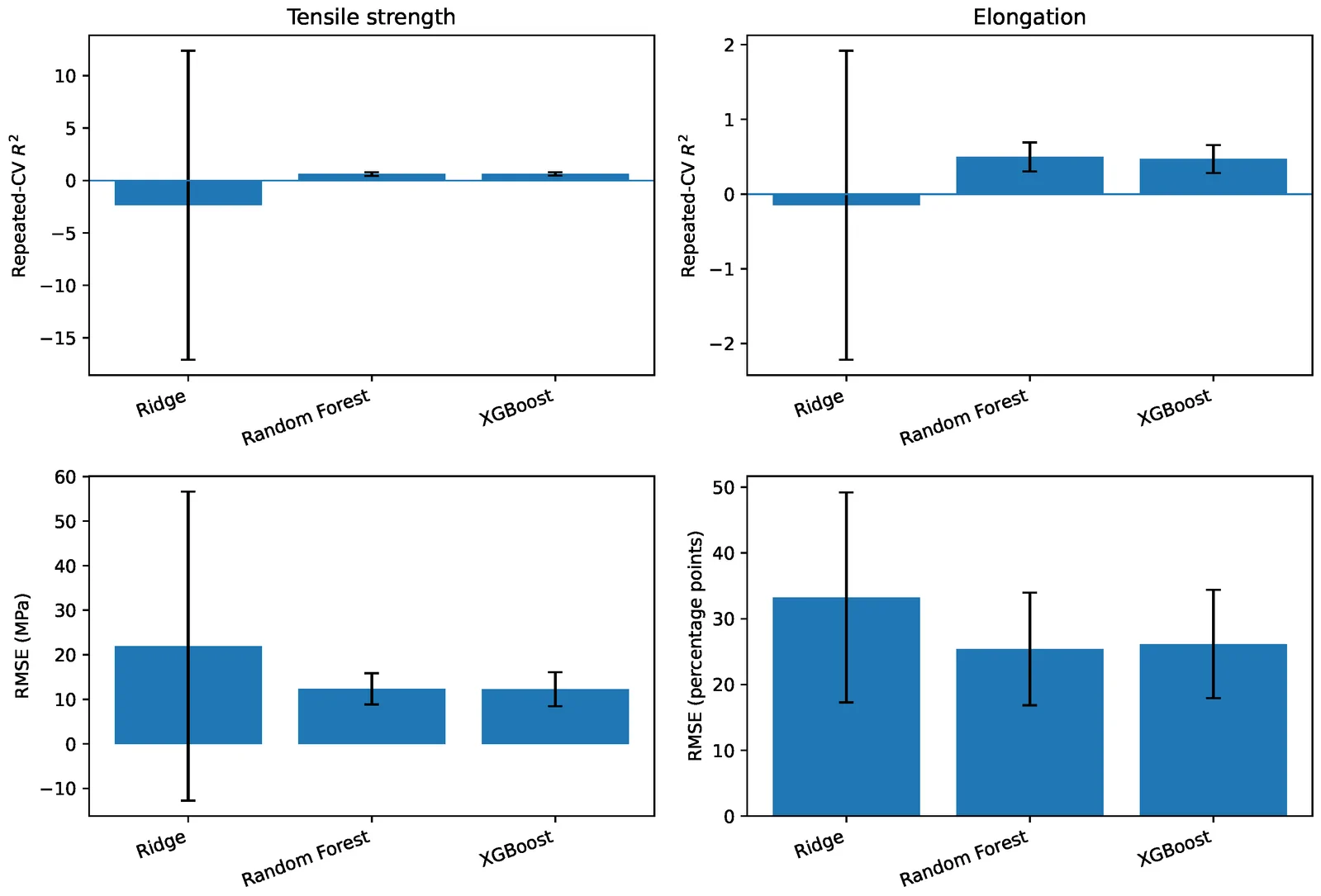
Repeated five-fold cross-validation performance. Bars show means and error bars show one standard deviation across 50 test-fold evaluations. The dispersion is part of the result and demonstrates why a single hold-out score is insufficient for this dataset.

Table 1 and Figure 2 replace the single 80/20 evaluation. For tensile strength, random forest and XGBoost produced similar mean performance: *R*^2^ = 0.617 ± 0.174 and 0.628 ± 0.150, respectively. Their RMSE values differed by only 0.08 MPa (12.35 versus 12.27 MPa), which is less than 1% of either mean RMSE and far below the fold-to-fold standard deviations. Thus, the revised analysis does not support a claim of clear XGBoost superiority. For elongation, random forest had the larger mean *R*^2^ (0.498 versus 0.470) and lower mean RMSE (25.41 versus 26.15 percentage points), again with overlapping resampling variability.

The ridge baseline was less stable, with mean *R*^2^ = −2.35 for tensile strength and −0.15 for elongation. Large negative values occurred in folds containing tail observations, consistent with multicollinearity, sparse additive occurrence, nonlinear interactions, and the limited sample-to-feature ratio. The linear baseline therefore does not resolve the overfitting problem; instead, it demonstrates that a global linear composition-property map is inadequate for these data.

### 3.3. Residual and Learning-Curve Diagnostics

**Figure 3:**
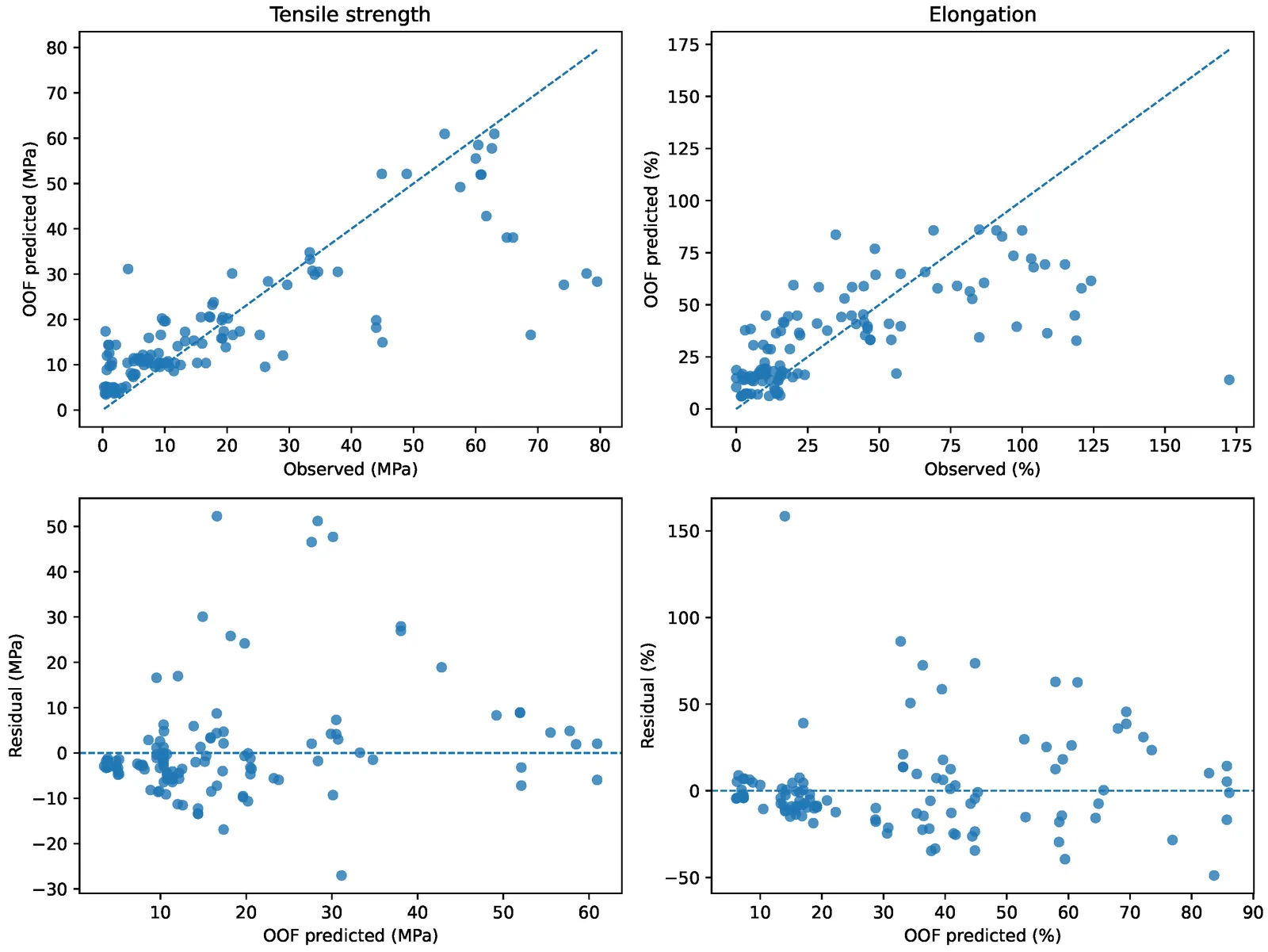
Out-of-fold XGBoost diagnostics from a deterministic shuffled five-fold split. Upper panels compare observed and predicted values; lower panels plot residuals against predictions. These plots are diagnostic and are not independent of the repeated-CV analysis.

Figure 3 shows out-of-fold predictions from one deterministic shuffled five-fold split for the regularized XGBoost model. The corresponding aggregate *R*^2^ values were 0.654 for tensile strength and 0.451 for elongation. Tensile residuals remain largest in the high-strength tail, while elongation predictions contract toward the densely sampled central region. This behavior is consistent with sparse coverage rather than specification-level prediction.

**Figure 4:**
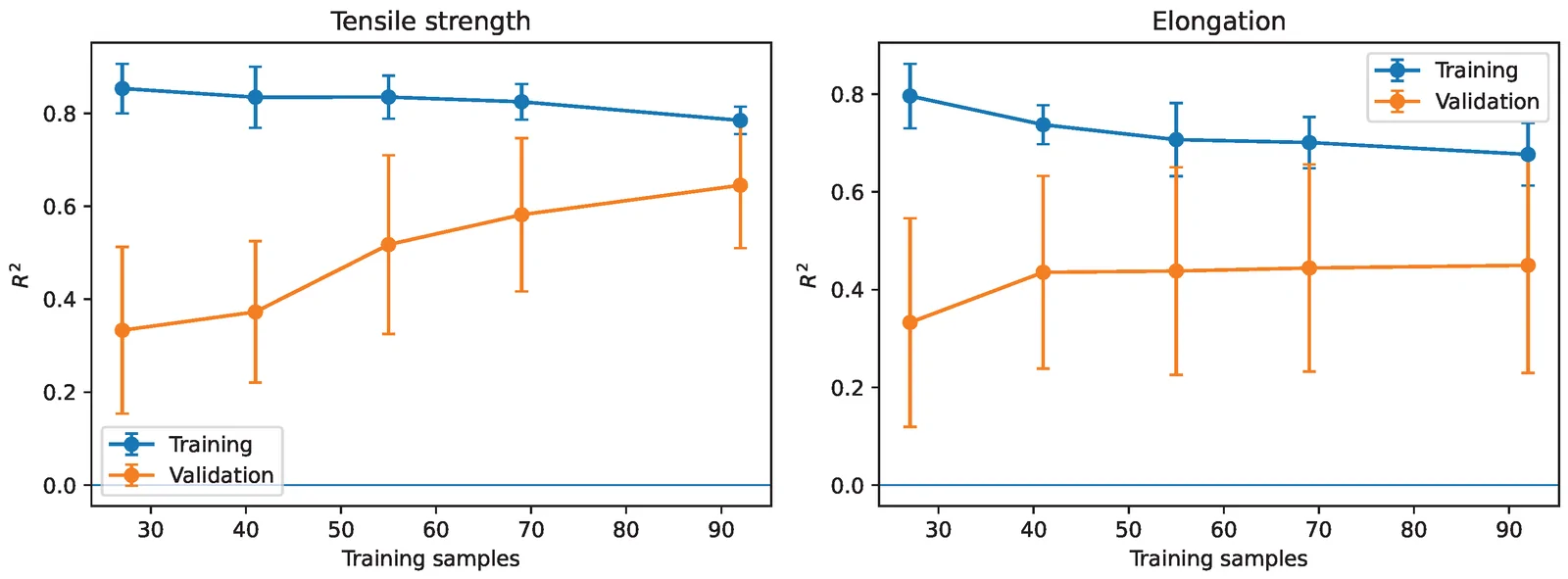
Learning curves for the regularized XGBoost model using five-fold cross-validation. Error bars show one standard deviation across folds. The remaining training-validation gaps indicate that model uncertainty is not eliminated at the available sample size.

Learning curves in Figure 4 show a persistent training-validation gap. For tensile strength, mean validation *R*^2^ increased from 0.35 with 27 training samples to 0.66 with 92, while training *R*^2^ decreased from 0.91 to 0.84. This trajectory indicates that additional data are likely to improve generalization. For elongation, validation *R*^2^ remained near 0.45 at the largest training size while training *R*^2^ remained about 0.75, indicating residual variance and descriptor insufficiency rather than a solved learning problem.

### 3.4. SHAP Attribution and Physical Interpretation

**Figure 5:**
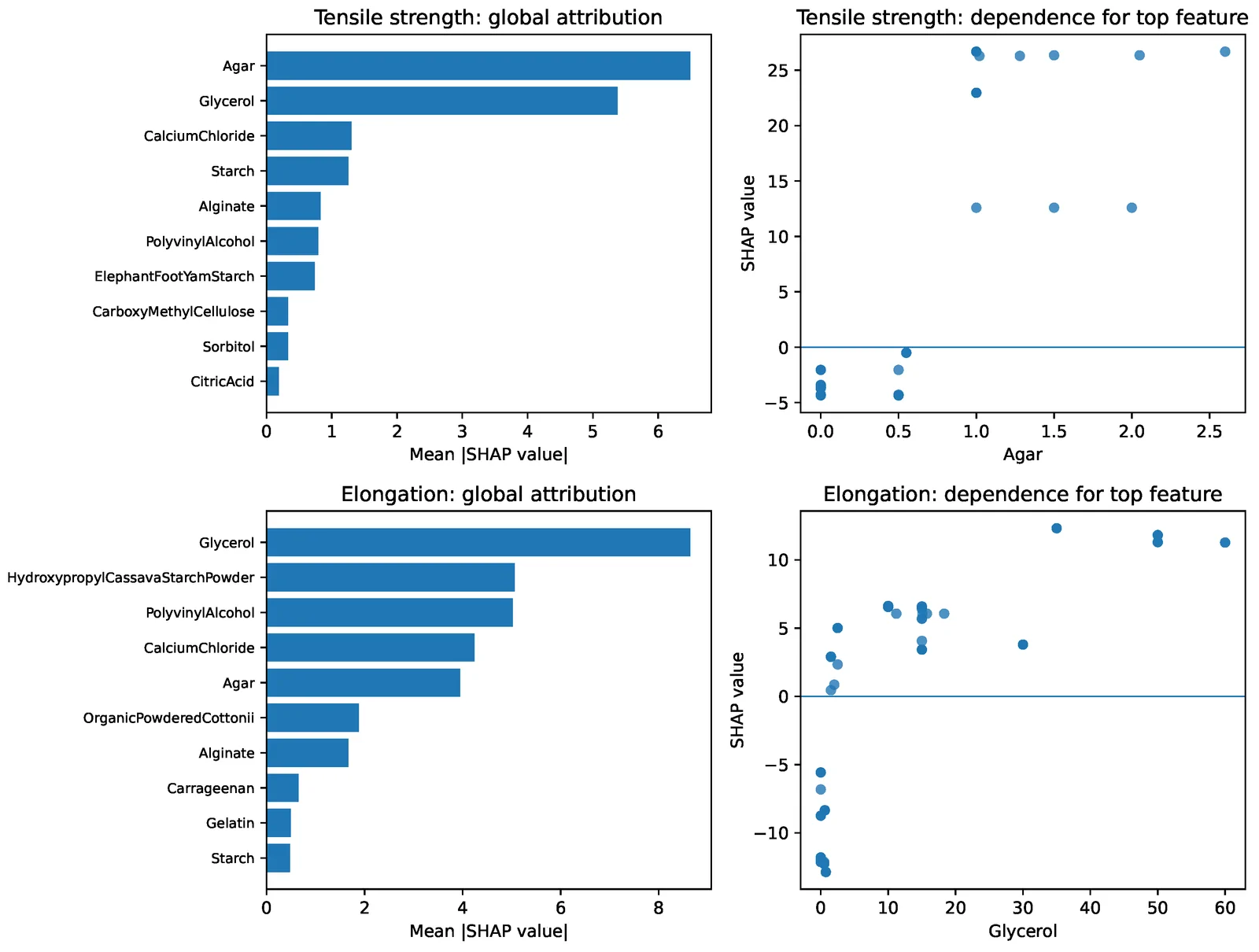
SHAP diagnostics for XGBoost models fitted to the full dataset after validation. Left panels show the ten largest mean absolute SHAP values; right panels show dependence for the leading feature of each target. Attributions describe the fitted model and are not causal effects.

Figure 5 provides model-attribution diagnostics. Agar had the largest mean absolute SHAP contribution for tensile strength, followed by glycerol, while glycerol was the leading attribution for elongation. These results are consistent with known mechanisms but cannot establish causality. Agar concentration is a proxy for network-forming polymer content, while glycerol can increase chain mobility and reduce cohesive interactions [4]. Calcium chloride, starch, polyvinyl alcohol, alginate, and other ingredients also appear among the leading attributions, indicating multicomponent interactions rather than a single-parameter response.

### 3.5. Ranked Seaweed Formulations

The five leading seaweed-containing formulations are listed in Table 2. Formulation 97 nearly matches the nominal tensile coordinate but reaches only 19.2% of the target elongation. Formulation 99 provides the largest elongation among the five, 108.79%, but remains 63.7% below 300% and its tensile strength is 49.9% below 15 MPa. Every candidate therefore deviates by more than 20% from at least one target coordinate, and none supports mechanical equivalence to LDPE.

## 4. Discussion

### 4.1. Plasticization, Moisture, and Storage Dependence

The strength–ductility trade-off has a direct physical basis. Neat polysaccharide films are stabilized by hydrogen bonding and can be brittle. Glycerol competes for intermolecular bonding sites, increases free volume, lowers effective glass-transition behavior, and increases segmental mobility; the usual macroscopic result is higher elongation with reduced stiffness and tensile strength [4]. This relation is not universal or monotonic because plasticizer compatibility, polymer molecular structure, water activity, concentration basis, and phase separation also matter.

Water is an additional plasticizer in agar-, alginate-, and carrageenan-rich networks. Relative humidity and storage conditions shift equilibrium moisture content and therefore alter mechanical response. A specimen conditioned at one humidity cannot be assumed mechanically exchangeable with a specimen tested at another humidity. Because humidity, storage duration, and conditioning protocol are not encoded in the source table, the current models cannot explicitly correct for these variables. Their effect is absorbed into unexplained residual variance or, more problematically, can be confounded with source study and formulation. This is why composition-only predictions are restricted to screening rather than qualification.

### 4.2. Multicomponent Interactions and Molecular Descriptors

Polymer identity alone is an incomplete descriptor. Agar contains agarose-rich fractions capable of thermoreversible network formation; alginate mechanics depend on mannuronic/guluronic sequence, molecular mass, and divalent-ion crosslinking; and carrageenan subtype and sulfate pattern alter gelation and hydration [1,2,3,18]. Equal nominal concentrations therefore do not imply equal network density or stress transfer.

Additives interact with these polymer networks. Calcium can reinforce alginate junctions while reducing extensibility; fillers can increase stiffness at low loading but create stress concentrations when agglomerated; oils may reduce water affinity while disrupting the continuous phase; and gelatin can form mixed protein-polysaccharide networks. The response surface is therefore coupled and non-additive. SHAP attribution is useful for exposing model dependence on these variables, but causal statements require controlled experiments in which composition, processing, and conditioning are independently varied.

Several physically important descriptors are absent: molecular mass, polydispersity, alginate M/G ratio, agarose fraction, carrageenan subtype, sulfate content, polymer grade, degree of substitution, dispersion quality, crosslink conversion, drying rate, residual moisture, thickness, and morphology. Increasing model complexity cannot recover information that is not represented in the predictors. The remaining learning-curve gap therefore provides evidence for both sample-size limitation and descriptor insufficiency.

### 4.3. Processing Dependence and Local Applicability

Film formation introduces a second unmodeled hierarchy. Solvent casting involves hydration, dissolution, mixing, degassing, casting, drying, and conditioning, all of which influence phase separation, entanglement, crystallinity, porosity, residual stress, and thickness gradients. Melt processing imposes different thermal and shear histories. Models trained on one mixture of processing routes may therefore fail when drying technology changes, when a new filler is introduced, or when a new polymer grade is used.

The present model is local in two senses. First, it is bounded by the composition and response support of the 115 records; the 300% elongation target lies outside that support. Second, random row-wise cross-validation does not test transfer to a new paper or laboratory when multiple related formulations originate from the same source. Grouped validation by source paper is required to quantify that transfer, but source identifiers are not available in the supplied dataset. This limitation is now explicit rather than hidden in a single random split.

### 4.4. Novelty Relative to the Source Dataset

The source article [8] used the same underlying records and related tree-based algorithms to predict composition and mechanical properties. The present study therefore does not claim novelty from dataset creation or algorithm choice. Its distinct technical contribution is a decision-focused secondary analysis: a declared two-property distance metric, a seaweed-only candidate ranking, quantitative demonstration that the adopted LDPE elongation target is outside the data domain, repeated resampling to quantify score instability, a regularized linear baseline, diagnostic learning/residual/SHAP analyses, and an experimental-validation pathway. This is a methodological reframing and risk-bounding contribution, not a new-material discovery claim.

### 4.5. LDPE Benchmark and Experimental Qualification

The term “LDPE-like” must be bounded by grade, thickness, processing direction, conditioning, and test method. LDPE film properties vary with branching, crystallinity, molecular-weight distribution, orientation, draw ratio, and processing. The adopted 15 MPa/300% point is therefore a nominal screening coordinate, not a universal LDPE specification. Sharing an ultimate tensile strength does not imply equivalent stress–strain behavior, toughness, creep, tear resistance, or failure mode.

Prospective qualification requires matched experiments. At a minimum, candidate films and a named commercial LDPE control should be produced or procured at matched thickness and tested after controlled conditioning using a declared tensile protocol. Replicate independent film batches, randomized specimen extraction, Young’s modulus, full stress–strain curves, toughness, tear/puncture response, water-vapor and oxygen transmission, seal strength, thermal transitions, dimensional stability, aging, and humidity sensitivity should be measured. Formulations that cannot form continuous films or that fail during drying should remain in the dataset as feasibility outcomes rather than being silently excluded.

### 4.6. Implications for Future Materials Informatics

A stronger next-stage workflow would link provenance-aware data curation to grouped or leave-one-study-out validation, uncertainty estimation, and prospective experiments. Source DOI, formulation basis, original units, polymer grade, thickness, conditioning, test speed, gauge length, drying conditions, and uncertainty should be retained for every row. Physics-informed descriptors such as plasticizer-to-polymer ratio, total solids, hydrophilic/hydrophobic fraction, ionic-crosslinker proxy, and filler fraction may improve transfer if concentration bases are normalized reliably.

The intended use is an iterative model-experiment loop: the model ranks candidates within an applicability domain; experiments test them under controlled conditions; failed and successful outcomes update the dataset; and the model is retrained. This use is consistent with materials-informatics frameworks in which machine learning prioritizes experiments rather than certifies materials autonomously [5,6,7,10].

## 5. Conclusions

The revised analysis does not support the original “inverse design” framing or a claim of LDPE-equivalent candidates. Repeated five-fold cross-validation shows moderate, variable predictive performance from composition-only tree ensembles: mean *R*^2^ values were approximately 0.62 for tensile strength and 0.47–0.50 for elongation, with substantial fold-to-fold dispersion. A regularized linear baseline was less stable, confirming that simple global linearity does not adequately describe the available composition-property relations. Reducing XGBoost depth and adding regularization limits model complexity but does not remove data scarcity, source heterogeneity, or omitted-variable bias.

The 300% elongation screening target lies outside the dataset: the observed maximum is 172.475%, 42.5% below the target, while the target is 73.9% above the observed maximum. All five ranked seaweed formulations deviate by more than 20% from at least one target coordinate. The model can therefore be used only to prioritize experiments within the observed domain. Claims of substitution require provenance-aware validation and controlled fabrication and testing against a named LDPE reference under matched conditions.

## Figures and Tables

**Table 1:** Repeated five-fold cross-validation results (10 repeats; 50 test-fold evaluations). Values are mean ± standard deviation across folds.

Target	Model	*R* ^2^	MAE	RMSE
Tensile strength	Ridge	−2.351 ± 14.733	11.00 ± 10.84 MPa	21.95 ± 34.68 MPa
Tensile strength	Random forest	0.617 ± 0.174	8.41 ± 1.76 MPa	12.35 ± 3.52 MPa
Tensile strength	XGBoost	0.628 ± 0.150	7.67 ± 1.95 MPa	12.27 ± 3.84 MPa
Elongation	Ridge	−0.148 ± 2.067	19.21 ± 6.66 pp	33.25 ± 15.97 pp
Elongation	Random forest	0.498 ± 0.194	16.53 ± 3.84 pp	25.41 ± 8.57 pp
Elongation	XGBoost	0.470 ± 0.188	17.41 ± 3.75 pp	26.15 ± 8.24 pp

**Table 2:** Selected seaweed-derived formulations and deviations from nominal screening coordinates.

ID	Matrix	σ (MPa)	ϵ (%)	Δσ (%)	Δϵ (%)
97	Carrageenan	15.21	57.52	+1.4	−80.8
99	Carrageenan	7.51	108.79	−49.9	−63.7
68	Agar/starch	9.20	85.00	−38.7	−71.7
60	Alginate/gelatin	9.00	85.00	−40.0	−71.7
70	Agar/starch	10.50	69.00	−30.0	−77.0

## Data Availability

The data presented in this study are available upon reasonable request from the corresponding author.
